# Post-war Azerbaijan: Burnout in mental health professionals working with war-affected populations

**DOI:** 10.1192/j.eurpsy.2023.1851

**Published:** 2023-07-19

**Authors:** R. Aliyeva, G. Guliyeva, F. İsmayilov

**Affiliations:** 1 Psychosocial Rehabilitation; 2 Director, National Mental Health Center of the Ministry of the Health, Baku, Azerbaijan

## Abstract

**Introduction:**

Burnout is a result of emotional exhaustion and lack of motivation after prolonged exposure to chronic emotional and interpersonal stressors at work. (Black et al. SAGE 2020, 609-611)

For the past decade, it has been one of the actual areas of research globally. (Ahola et al. Journal of affective disorders, 88, 2005; 55–62; Ahola et al. Journal of affective disorders,104,2007; 103–110, Shirom, A. Work Stress, 19, 2005; 263–270, Toker et al. The Journal of Applied Psychology, 97, 2012; 699–710.) However, a small number of studies exploring burnout have been conducted in Azerbaijan.

The requirement for supportive interventions for workers with an aim to improve the quality of care and prevention of burnout related to health and sociocultural problems such as difficulties in their personal life, job-shifts has led us to conduct this study.

Above all, the Second Karabakh war that took place in 2020 increased the population of patients that are in urgent need of psychiatric and psychological support.

**Objectives:**

The purpose of this study is to examine the level of burnout among all mental health workers who have been closely working with veterans and families of deceased soldiers. Similarly, the study is intended to evaluate the impact of various factors, such as secondary traumatic stress, effort-reward imbalance, and socio-demographic variables leading to burnout.

**Methods:**

The study design is cross-sectional. 22-item Maslach Burnout Inventory (MBI) is used to measure the level of burnout. Intrusion, avoidance, and arousal symptoms triggered by indirect exposure to traumatic events are evaluated by the 17-item Secondary Traumatic Stress Scale (STSS). 22-item Measurement of the effort-reward imbalance (ERI) is used to define the level of effort, reward, and over-commitment. Demographic questionnaires consist of age, sex, marital status, professional background, years of employment, workload in hours.

**Results:**

The sample size has been estimated as 200 participants. Associations between occupational exhaustion, depersonalization, personal accomplishment assessment, intrusion, avoidance, arousal, effort, esteem, job promotion, job security, overcommitment and professional background, workload in hours will be explored in the current study.

**Conclusions:**

The findings upraised will promote elaborating personalized approaches toward burnout prevention treatment.

**Disclosure of Interest:**

None Declared

